# An updated guide to hair follicle stem cell markers and changes in their expression with aging

**DOI:** 10.1016/j.xjidi.2026.100459

**Published:** 2026-02-12

**Authors:** Theebah Sellathurai, Denise L. Gay, Stéphane Commo, Gilles Lemaître, Nicolas O. Fortunel

**Affiliations:** 1 Paris-Saclay University, Gif-sur-Yvette, France; 2 CEA, Laboratory for Cutaneous Regeneration and Radiopathologies (LR2C), Evry, France; 3 CEA, François Jacob Institute of Biology (IBFJ), Department of Cellular and Molecular Radiobiology (DRCM), Fontenay-aux-Roses, France; 4 INSERM UMR Genetic Stability, Stem Cells and Radiation, Fontenay-aux-Roses, France; 5 L'Oréal Research and Innovation, Aulnay sous bois, France; 6 DLGBiologics, Paris, France; 7 Evry Paris-Saclay University, Évry-Courcouronnes, France

**Keywords:** Aging, Hair biology, Genomics, Keratinocyte biology, Stem cells

## Abstract

The availability of identification markers is a major expectation in the field of stem and progenitor cell biology, whether to decipher the hierarchy of these cellular compartments or to isolate these cells for use in tissue reconstruction and regeneration approaches. Epithelial hair follicle stem cells (HFSCs) constitute a well-established model of multipotent tissue stem cells. Knowledge of relevant HFSC phenotypes is crucial for their identification and manipulation for therapeutics, including hair regeneration in patients with alopecia and skin engineering after injury. In this review, we provide a detailed review of murine and human HFSC markers, drawing upon traditional studies that identify classic HFSC markers and highlighting single-cell RNA-sequencing studies that have greatly expanded our knowledge of HFSCs. Recently defined HFSC subsets with distinct marker expression, microRNAs that qualify as cycle-specific HFSC markers, and HFSC marker changes with aging are all discussed.

## Graphical abstract

**Figure:**
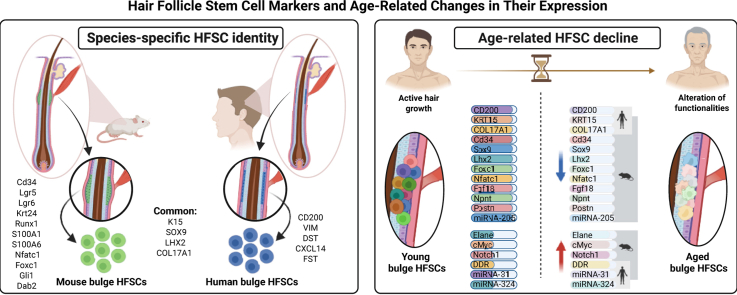

## Introduction

The biology of the epithelial stem cell (SC) populations (hair follicle SCs [HFSCs]) hosted within hair follicles (HFs) constitutes a crossroads where fundamental research and applied research fields intersect. At the basic research level, these cells serve as a pioneer model for deciphering the mechanisms and signals involved in the control of SC identity and fate, including stemness maintenance, self-renewal, and orientation toward differentiation (Cotsarelis, 2006; Fuchs et al, 2001; Kretzschmar and Watt, 2014). In terms of potential applications, they constitute potential cellular targets for antiaging strategies and regenerative approaches, including the prevention and/or reversal of alopecia (Mistriotis and Andreadis, 2013; Plikus, 2014) and skin repair (Ito et al, 2005). Knowing the markers that define these cells is crucial for their identification and study. In this review, we used the term “marker” to denote a molecular feature (protein, mRNA, microRNA [miRNA], or epigenetic mark) whose expression is predominant in a defined HFSC population compared with surrounding cells. These markers are not necessarily exclusive to the HFSCs and can be present in other epidermal compartments (eg, basal layer keratinocytes).

Hair miniaturization and subsequent hair loss (alopecia) are common features of murine and human chronological aging (Harrison and Bergfeld, 2009; Paus and Cotsarelis, 1999; Whiting, 2001). Because of their pivotal role in hair cycling, HFSCs have taken center stage in studies trying to understand the effects of aging on hair miniaturization and ultimately alopecia. Understanding age-related changes in established HFSC markers and how they impact HFSC biology is an essential step toward treatment of alopecia (Jang et al, 2023; Ji et al, 2017).

Single-cell RNA sequencing (scRNAseq) has dramatically broadened our knowledge of HFSCs. In particular, it has provided a highly sensitive means of validating described markers, including those specific to the hair cycle phase, and has provided candidates for future study (Chovatiya et al, 2021; Joost et al, 2016; Ober-Reynolds et al, 2023; Takahashi et al, 2020; Wu et al, 2022). It has revealed multiple subsets within the bulge. Recent examination of miRNAs (known as post-transcriptional regulators that modulate mRNA stability and translation) during telogen and telogen-to-anagen transition has provided insights into HFSC regulation and demonstrated that miRNAs can themselves serve as stage-specific markers (Ding et al, 2022).

In this review, we have examined recently available scRNAseq analyses to provide a list of validated HFSC markers, including phase-specific markers, for both mice and humans. To understand how aging impacts HFSCs, age-related changes in known and HFSC markers are addressed.

## The HF Cycling

**Figure 1:**
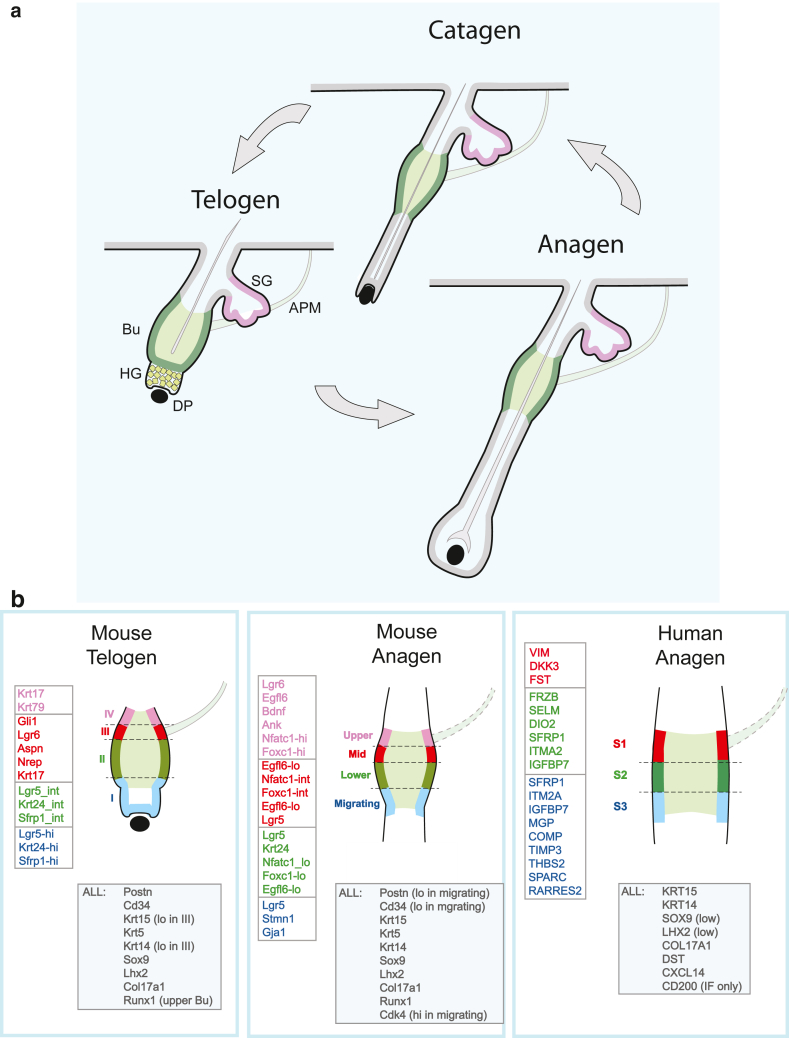
**Specificities of human and murine HFSC marker profiles.** (**a**) HF cycle. Distinct HF regions are labeled: SG for sebaceous gland, APM for arrector pili muscle, Bu for bulge, HG for hair germ, and DP for dermal papilla. The introduction provides details. (**b**) Expression of bulge region–dependent markers. Mouse telogen: based on findings from Joost et al (2016). Mouse anagen: based on findings from Chovatiya et al (2021). Human anagen: based on findings from Wu et al (2022). “ALL” refers to markers found in all regions. In human region S3, only high expressors have been shown. Wu et al (2022) provides details. APM dotted line indicates that the boundary is assumed. HF, hair follicle.

HF cycling, a phenomenon common to all mammals, is the process through which the entire lower follicle part is cyclically rebuilt (Cotsarelis, 2006; Driskell et al, 2011; Fuchs et al, 2001). It involves 3 phases: telogen (rest phase), anagen (growth phase), and catagen (lower follicle regression) (Figure 1a). Multiple studies have shown that HFSCs are responsible for rebuilding the lower HF with each hair cycle, ultimately giving rise to all lower follicular layers, including the new hair shaft productive compartment (Alonso and Fuchs, 2006; Cotsarelis, 2006; Paus and Cotsarelis, 1999). During telogen, HFSCs, in their niche environment termed the bulge and in a transitional structure directly below, termed the hair germ (HG), are in close proximity to a specialized appendage structure containing dermal fibroblasts (dermal papilla [DP]) (Figure 1a). Complex signaling between these structures during anagen onset initiates HFSC activation and the beginning of a growth period (anagen onset). During anagen, HFSCs undergo a highly regulated program of activation, proliferation, and ultimately differentiation to build the entire lower follicle. After growth cessation, the lower follicle undergoes regression, resulting in complete lower follicle loss and eventual shedding of the hair shaft (catagen). Catagen is followed by a period of rest (telogen), during which the HFSCs prepare for a next cycle (anagen onset).

HFSCs are organized into distinct subpopulations with specific spatial localizations and functional roles during the hair cycle. In this paper, we delineate the distinct HFSC subpopulations in both mouse and human HFs and adopt a consistent nomenclature when discussing their markers. Some markers are predominantly associated with particular HFSC subpopulations, and their expression varies depending on the stage of the hair cycle (Figure 1b). In the mouse, these HFSC subpopulations can be defined precisely according to their position along the follicle axis. During telogen, compartment I corresponds to the HG, located above the DP; compartment II corresponds to the lower telogen bulge, situated just above the HG; compartment III corresponds to the middle bulge; and compartment IV corresponds to the upper bulge, located near the arrector pili muscle (APM). During murine anagen, compartment I is referred to as the “migrating” population, whereas compartments II, III, and IV are designated as “lower,” “middle,” and “upper” bulge compartments, respectively. In human anagen HFs, a true ‘bulge’ does not exist, but analogous layers, containing cells with equivalent markers, are defined as S1, S2, and S3, from top to bottom, with S1 adjacent to the APM. The specific compartment will be referred to for markers that are specific to that compartment.

HFSCs have received considerable attention from the regenerative biology community for their regenerative and multipotent capacities. Research efforts have focussed on using HFSCs to rebuild follicles (and skin) in patients with alopecia and to repair skin after skin trauma events. In this article, we examine the most recent results to identify and validate HFSC markers in mice and humans and assess how these markers change with aging.

## Markers Common to Murine and Human HFSCs

### Keratin 15

Keratins, subclassified as cytokeratins and hair keratins, are intermediate filament proteins that ensure structural integrity of epithelial cells (Jacob et al, 2018). They have been credited with a wide range of associated functions, including transcription regulation, adhesion, migration, and polarity. Keratin (K)15 is a type I cytokeratin. It remains a quintessential HFSC marker in both humans and mice (Braun et al, 2003; Kloepper et al, 2008; Liu et al, 2003). Sorting experiments showed that K15-EGFP+ HFSCs were typically quiescent, a hallmark feature of SCs (Morris et al, 2004). Lineage tracing revealed their ability to regenerate all HF cell types during hair cycling (Morris et al, 2004). The SC status of K15^+^ HF epithelial cells was also documented using a transgenic mouse model expressing the suicide gene herpes simplex virus thymidine kinase under the control of the K15 promoter. This system enabled the selective destruction of K15^+^ bulge cells, resulting in progressive HF loss (Ito et al, 2005). In human HFs, K15 staining also delineates the bulge-like region (Kloepper et al, 2008; Purba et al, 2014).

Complementary immunofluorescence (IF) and recent scRNAseq results verify K15 expression throughout the human bulge-like area during anagen and the mouse bulge during both telogen and anagen phases (Table 1 and Figure 1b) (Chovatiya et al, 2021; Morita et al, 2021; Takahashi et al, 2020; Wu et al, 2022).

**Table 1:** HFSC Markers Verified by scRNAseq Analysis during Mouse HF Anagen and Telogen Phases or Human HF Anagen Phase

Marker	Mouse		Human
	Anagen	Telogen	Anagen
Human and mouse			
K15	√ (Chovatiya et al, 2021)	√ (Joost et al, 2016)	√ (Takahashi et al, 2020; Wu et al, 2022)
SOX9	√ (Chovatiya et al, 2021)	√ (HG) (Ge et al, 2020; Joost et al, 2016)	√ (low) (Ober-Reynolds et al, 2023; Wu et al, 2022)
LHX2	√ (Chovatiya et al, 2021)	√ (Ge et al, 2020; Joost et al, 2016; Zhang et al, 2021)	√ (low) (Ober-Reynolds et al, 2023; Wu et al, 2022)
COL17A1	√ (Chovatiya et al, 2021)	√ (Joost et al, 2016)	√ (Wu et al, 2022)
Phase specific			
SFRP1		√ (I and II) (Ge et al, 2020; Joost et al, 2016; Zhang et al, 2021)	√ (S2) (Ober-Reynolds et al, 2023; Wu et al, 2022)
DKK3		√ (Ge et al, 2020; Joost et al, 2016)	√ (S1 and S2) (Ober-Reynolds et al, 2023; Wu et al, 2022)
Mouse			
CD34	√ (Chovatiya et al, 2021)	√ (Ge et al, 2020; Joost et al, 2016; Zhang et al, 2021)	√ (bulge [scRNAseq] and HG [IF]) (Mohammadi et al, 2021; Wu et al, 2022)
LGR5	√ (low in upper-bulge) (Chovatiya et al, 2021)	√ (I and II + HG) (Ge et al, 2020; Joost et al, 2016; Zhang et al, 2021)	√ (HG and lower bulge) (Ober-Reynolds et al, 2023; Takahashi et al, 2020)
POSTN	√ (low in mid-bulge) (Chovatiya et al, 2021)	√ (Ge et al, 2020; Joost et al, 2016; Zhang et al, 2021)	
NPNT	√ (Chovatiya et al, 2021)	√ (Ge et al, 2020; Joost et al, 2016)	
K24	√ (lower-bulge) (Chovatiya et al, 2021)	√ (I and II) (Ge et al, 2020; Joost et al, 2016; Zhang et al, 2021)	
RUNX1	√ (Chovatiya et al, 2021)	√ (III) (Joost et al, 2016)	
S100A4	√ (Chovatiya et al, 2021)	√ (Ge et al, 2020; Joost et al, 2016; Zhang et al, 2021)	
S100A6	√ (Chovatiya et al, 2021)	√ (Joost et al, 2016)	
LGR6	√ (upper-bulge) (Chovatiya et al, 2021)	√ (III) (Joost et al, 2016)	
Phase specific			
FOXC1	√ (highest in upper-bulge) (Chovatiya et al, 2021)		
NFATC1	√ (high in upper-bulge) (Chovatiya et al, 2021)	√ (III) (Joost et al, 2016)	√ (low) (Wu et al, 2022)
GLI1		√ (III) (Joost et al, 2016)	
DAB2		√ (upper) (Joost et al, 2016)	
Human			
VIM			√ (S1 and S2) (Wu et al, 2022)
IRF1			√ (Ober-Reynolds et al, 2023)
CD200			√ (IF only) (Ober-Reynolds et al, 2023; Wu et al, 2022)
DST			√ (Wu et al, 2022)
CXCL14			√ (Takahashi et al, 2020; Wu et al, 2022)

Abbreviations: DST, dystonin; HF, hair follicle; HG, hair germ; IF, immunofluorescence; K, keratin; NPNT, nephronectin; scRNAseq, single-cell RNA sequencing; VIM, vimentin.

Locations I, II, and II during mouse telogen and S1 and S2 during human anagen correspond to bulge area segments as depicted in Figure 1b.

### K19

As described for K15, the expression of K19 was reported to be restricted to the outer root sheath (ORS) in human HFs, and it was weakly detected in the epidermis (Stasiak et al, 1989). More precisely, K19 expression was detected in the bulge area and colocalized with ITGB3^bright^/ITGB1^bright^ keratinocytes (Michel et al, 1996). Interestingly, in mouse, K19^+^ keratinocytes were shown to be noncycling, consistent with a HFSC status (Michel et al, 1996). In addition, in human HFs, K19 expression colocalized with K15 within the bulge area but also extended downward throughout the entire ORS basal layer, from the bulge to the bulb (Lyle et al, 1998). Of note, human HFs were shown to contain 2 distinct reservoirs of K19^+^ keratinocytes, within upper and lower regions (Commo et al, 2000). In human HFs, expression of K19 was reported to be stronger within the proximal bulb ORS than within the bulge region (Purba et al, 2015). Recently, scRNAseq analysis located K19 expression in cells of the bulge region of human HFs (Takahashi et al, 2020).

### SOX9

The SOX9 (SRY-Box transcription factor 9) is known as a regulator of chondrocyte differentiation (Lefebvre et al, 2019), skeletal development (Lee and Saint-Jeannet, 2011), HF morphogenesis, and differentiation (Vidal et al, 2005).

In the mouse, SOX9 is found in a label-retaining and thus quiescent subset of embryonic hair placode cells, later giving rise to bulge HFSCs. Conditional ablation of *Sox9* prior to placode formation results in developmental blockade of HF and sebaceous glands morphogenesis and interfollicular epidermis (IFE) wound healing defects (Nowak et al, 2008). In the adult, SOX9 colocalizes with CD34 in the mature HF bulge (Kadaja et al, 2014; Vidal et al, 2005), and conditional ablation revealed that *Sox9* is also required for adult HFSC maintenance (Kadaja et al, 2014). Its loss results in eventual HFSC loss and HF cycle arrest (Kadaja et al, 2014). scRNAseq analyses have confirmed *Sox9* expression in the murine HF bulge during anagen and telogen phases (Table 1).

In human HFs, SOX9 is more broadly expressed in the HF. Using IF analysis, SOX9^+^ cells have been detected throughout the ORS, with the highest abundance in the human HF region related to the mouse sub-bulge (Purba et al, 2015). A small subpopulation was shown to coexpress K15, LHX2, and SOX9 at the border between the bulge and sub-bulge regions (Purba et al, 2015). scRNAseq results confirm low *SOX9* expression within the bulge but also expression outside the bulge (Table 1).

### LHX2

The LHX2 (LIM homeobox protein 2) belongs to a transcription regulator family carrying a cysteine-rich zinc-binding domain that has been implicated in a broad range of developmental contexts.

In the mouse, during embryogenesis, LHX2 expression was reported in early hair placodes and at the leading front of invaginating HGs and pegs (Tomann et al, 2016). In mouse adult HFs, IF analysis showed that LHX2 expression localizes to the bulge and secondary HG (Mardaryev et al, 2011; Rhee et al, 2006). Conditional knockout (KO) of the *Lxh2* gene in HFs revealed increased proliferation in the bulge and more rapid HF cycling (Rhee et al, 2006). Later work revealed that LHX2 activates a variety of cytoskeletal and adhesion genes within HFSCs and that its loss resulted in broad changes in HFSC arrangement within the bulge and in overall bulge architecture, implying that HFSCs manage their niche environment through LHX2 expression (Folgueras et al, 2013). scRNAseq has verified the expression of this marker during both telogen and anagen (Table 1).

In human HFs, LHX2 expression has been conversely reported either within human HF bulge and HG (Mardaryev et al, 2011) or, alternatively, primarily outside the bulge and within the sub-bulge and proximal bulb ORS (Kloepper et al, 2008; Purba et al, 2015). scRNAseq results support the former observation, indicating that *LHX2* expression is restricted to the human anagen bulge (Table 1).

### COL17A1

Transmembrane collagen 17 is a structural component of hemidesmosomes, multiprotein complexes that attach the cell to an adjacent membrane (Franzke et al, 2005). *COL17A1* gene refers to the collagen 17 alpha chain. In the adult, COL17A1 is commonly expressed by interfollicular basal epithelial cells and by HFSCs in the bulge. Function(s) include maintenance of the niche architecture, but other functions, including enhanced competitiveness for SCs with higher collagen 17 levels (Liu et al, 2019) and regulation of SC proliferation (Watanabe et al, 2017), have been identified at least in the IFE. These additional functions remain to be examined in HFSCs. Its loss during aging is directly linked to the aging process in skin and in HFs (see below).

*COL17A1* expression, previously identified in the HF bulge through IF analysis, has been verified by scRNAseq in both mouse and human HFSCs during at least telogen and anagen or anagen, respectively (Table 1).

### WNT inhibitors

WNT activity is crucial for expansion and differentiation of HFSCs within the HG during early anagen. In contrast, its activity is strictly regulated in the bulge to ensure that HFSCs retain a quiescent undifferentiated status throughout most of the cycling period (Choi et al, 2013; Lien et al, 2014). Secreted frizzled-related protein family members and Dickkopf-related protein family members are well-recognized inhibitors of WNT activity by binding either WNT ligands and WNT receptors, respectively (Liu et al, 2022). Members of both families have recently been identified in mouse telogen-phase HFSCs and human anagen HFSCs through scRNAseq analyses (Table 1). Both or related family members are likely expressed within the bulge throughout the hair cycle, although additional work will be needed to verify this. WNT family members have been implicated in HFSC aging (details are provided in the section on changes in the expression of markers with aging).

## Markers that Define Murine HFSCs

### CD34

The transmembrane sialomucin protein CD34, a well-known marker used for the selection of hematopoietic stem and progenitor cells, functions as a cell–cell adhesion factor (Ohnishi et al, 2013). Previous work has demonstrated that the CD34 marker is detected within the murine telogen bulge but not the HG (Greco et al, 2009; Nowak and Fuchs, 2009; Trempus et al, 2003). Recent scRNAseq has validated the expression of this marker throughout the murine bulge during both telogen and anagen phases (Figure 1b and Table 1).

In human anagen HFs, the isthmus, bulge, and infundibulum regions of the ORS have traditionally been found negative for CD34, whereas expression was noted in the lower ORS (Garza et al, 2011; Inoue et al, 2009; Ohyama et al, 2006). Recent results remain contradictory. One scRNAseq study described *CD34* mRNA expression restricted to bulge cells (Wu et al, 2022), and another study strictly associated CD34 protein (detected by IF) with the HG (Mohammadi et al, 2021) (Table 1).

### LGR5

The leucine-rich repeat-containing receptor family belongs to the R-spondins-G protein-coupled, 7-transmembrane receptor superfamily, which constitutes the largest family of mammalian membrane receptors (Barker et al, 2013; Smith et al, 2016). R-Spondins are the ligands for LGR5, and their coassociation promotes WNT activity in a variety of cells (Smith et al, 2016). Functionally, LGR5^+^ keratinocytes were shown to ensure the reconstitution of fully formed HFs with a high efficiency after transplantation onto the backs of nude mice (Jaks et al, 2008). However, diphtheria-toxin–mediated cell ablation of murine HF LGR5^+^ cells only temporarily abrogated hair regeneration (Hoeck et al, 2017). These combined results suggest that LGR5+ cells likely represent ‘primed’ HFSCs, which do not overlap with or replace quiescent HFSCs.

In earlier studies, LGR5 expression was reported within the lower ORS region of mouse HFs during anagen and catagen (Jaks et al, 2008) and the lower bulge during telogen (Greco et al, 2009; Jaks et al, 2008). Of note, LGR5 expression was also reported in mouse HFs to partially overlap with that of CD34 in the bulge area of telogen HFs (Hoeck et al, 2017; Jaks et al, 2008). Recent scRNAseq results have placed *Lgr5* in the lower bulge during both telogen and anagen (Table 1 and Figure 1: compartment “II” and “lower”) (Chovatiya et al, 2021; Joost et al, 2016), and additional reports have shown it in the telogen HG as well (Ge et al, 2020; Zhang et al, 2021). This location supports its role as a marker of later ‘primed’ HFSCs.

In human HFs, identification of LGR5 expression in the bulge has remained controversial. Its expression in the bulge has recently been demonstrated by IF staining (Polkoff et al, 2022), but scRNAseq analyses place it within the HG (Ober-Reynolds et al, 2023).

### Proteins in APM attachment, POSTN, and nephronectin

The smooth muscle, called APM, attaches to the HF bulge region and confers hair erection (piloerection) as well as sympathetic nerve support (Shwartz et al, 2020). Several extracellular matrix (ECM) proteins secreted by HFSCs are implicated in APM attachment, including POSTN (periostin) and nephronectin (NPNT).

POSTN, associated with adhesion primarily in tendons/ligaments, has recently been shown to have important roles in SC-mediated tendon and bone repair (Duchamp De Lageneste et al, 2018; Wang et al, 2021). In the adult HF, Fujiwara et al (2011) observed high expression of periostin in bulge HFSCs during both telogen and anagen through IF analysis. scRNAseq validated its expression during both phases (Table 1), and examination for region-specific expression has suggested higher levels within the anagen upper/mid bulge (Chovatiya et al, 2021) and, conversely, higher levels within the telogen lower bulge (Fujiwara et al, 2011; Joost et al, 2016), although low *Postn* expression was also observed in all other regions at both time points. Localization to the APM attachment site remains inconclusive, although Joost et al (2016) found that related ECM protein EGFL6, also postulated to attach to APM, was expressed throughout the telogen bulge.

NPNT, another primary candidate for bulge–APM anchorage, localizes to the basement membrane of the bulge, hair bulb, and APM in adult telogen and anagen HFs (Fujiwara et al, 2011). In *Npnt*-KO HFs, APM attachment was lost in a subset of follicles, whereas compensatory overexpression of EGFL6 was observed in the other HFs, leading to a shift of APM attachment to this EGFL6-positive zone (Fujiwara et al, 2011). scRNAseq has validated *Npnt* gene expression within HFSCs during both anagen and telogen (Table 1), although its exact distribution within bulge subregions has yet to be determined.

### K24

K24 is a cytokeratin-like protein belonging to the cytokeratin I family. Although historically considered a mouse skin keratin, recent work reveals very low expression of K24 in human epidermis (Ehrlich et al, 2019).

In murine HFs, scRNAseq analyses have verified *K24* expression within bulge HFSCs during both anagen and telogen (Table 1). Its bulge expression appears largely confined to the mid-lower bulge during both phases (Figure 1b: compartment “II” and “lower”).

### RUNX1

The RUNX1 (RUNX family transcription factor 1) forms a complex with the cofactor CBFB and is a master regulator of hematopoietic SC differentiation and specification (Kumano and Kurokawa, 2010).

Lineage tracing in *Runx1*-LacZ knock-in mice revealed Runx1 expression in the lower bulge and HG at the telogen phase, which was consistent with previous data on Runx1 expression localized by IF (Lee et al, 2014; Osorio et al, 2008). Its expression was found downregulated in HG cells as they initiated downgrowth to create matrix, whereas it remained stable within the bulge during anagen progression (Hoi et al, 2010; Lee et al, 2014). Furthermore, in *Runx1*-CreER knock-in mice induced during the catagen phase, X-gal–labeled cells were detected in both the upper and lower bulge. These *Runx1*-expressing cells are indeed derived from HFSCs because they are involved in the anagen bulb and the hair shaft formation during anagen (Scheitz et al, 2012). In conditional epithelial *K14*-driven *Runx1* KOs, transition to anagen was strikingly delayed, and HFSC proliferation during anagen onset was impaired, again demonstrating the importance of *Runx1* (Hoi et al, 2010).

scRNAseq has verified the expression of *Runx1* within bulge HFSCs during both telogen and anagen, although surprisingly, its expression has been allocated to upper rather than lower bulge during telogen (Figure 1b and Table 1), suggesting a possible increased stability of *Runx1* mature mRNA in this cell compartment. The lack of mature mRNAs in HG could result from regulatory mechanisms leading to Runx1 rapid shut down in dividing HG cells (Lee et al, 2014).

### S100A4 and S100A6

Proteins of the S100 family are characterized by a structure containing 2 EF-hand calcium-binding motifs (Santamaria-Kisiel et al, 2006). These calcium-binding proteins have a cytoplasmic and/or a nuclear localization and are involved in the regulation of several biological processes, including cell cycling and differentiation, in many cell types (Donato, 2003).

The S100 family members S100A4 and S100A6 are expressed in mouse HFs with distinct patterns according to hair cycle phases (Ito and Kizawa, 2001). In mouse HFs, S100A4 has been detected exclusively in the bulge area during all examined cycling phases, whereas S100A6 exhibited broader expression during telogen (bulge and HG) and anagen (bulge and downgrowing matrix) (Ito and Kizawa, 2001). Transcription of both *S100a4* and *S100a6* was reported to precede cell proliferation at anagen onset, indicating that both are associated with HFSC activation at the onset of follicle regeneration (Ito and Kizawa, 2001).

scRNAseq has validated the expression of both genes in mouse bulge HFSCs during anagen and telogen (Table 1).

### LGR6

The LGR6 (leucine-rich repeat-containing G-protein-coupled receptor 6) belonging to the G-protein–coupled receptor family, is an SC marker in many tissues, including taste buds, lung, mammary gland, and HFs. Studies revealed its early expression in the embryonic HG and final location in the adult HF upper bulge/infundibulum region (Snippert et al, 2010). Labeling of adult HF LGR6+ cells showed that they can give rise to sebaceous gland and interfollicular epithelium; however, these cells do not appear to contribute to hair cycling (ie, lower HF regeneration), suggesting that they are primarily involved in upper HF maintenance. They are also important for sustained long-term healing of the wounded IFE (Snippert et al, 2010). *Lgr6* expression has been verified in upper bulge HFSCs during both anagen and telogen (Table 1 and Figure 1b: compartment “III” and “upper”).

### FOXC1

FOXC1 (Forkhead box C1) is a member of the forkhead family of transcription factors, whose specific function has not yet been determined (Han et al, 2017; Wang et al, 2018).

Transcription factor FOXC1 is upregulated during early anagen and remains active in the HF bulge throughout the anagen phase (Lay et al, 2016; Wang et al, 2016). *K14*cre conditional KOs of *Foxc1* showed premature hair growth due to shortened telogen period, increased bulge HFSC proliferation throughout anagen (BrdU studies), and loss of the club hair with its accompanying original (old) bulge (Lay et al, 2016; Wang et al, 2016). An important role for FOXC1 is therefore to re-establish HFSC quiescence during early anagen. Downstream targets include *Nfatc1* and *Bmps*, both known to negatively control HFSC activation (Wang et al, 2016). Loss of *Foxc1* and *Nfatc1* during aging has important consequences for HF maintenance (details are provided in the section on aging).

In studies focusing on ‘old’ bulge loss in *Foxc1* KOs, it was shown that HFs could still form a bulge at the second telogen stage, but the old bulge did not remain anchored. Consequently, as the hair emerged, the entire old bulge, including its reserve HFSCs, was lost (Lay et al, 2016). Additional studies revealed that loss of the old bulge itself could marginally impact hair cycling (Lay et al, 2016).

### NFATC1

Nuclear factors of activated T cells constitute a transcription factor family that plays an important role in regulating immune responses (Serfling et al, 2000).

In mouse HFs, NFATC1 expression has been shown to coincide with CD34 expression in the bulge area and to persist throughout both anagen and telogen phases (Horsley et al, 2008). Loss of *Nfatc1* in conditional KOs phenocopied *Foxc1* KOs exhibiting a shortened telogen, increased HFSC proliferation within the anagen bulge, and loss of club hairs (Horsley et al, 2008; Lay et al, 2016). Furthermore, NFATC1 was shown to downmodulate HFSC proliferation in the bulge by repressing expression of cell cycle regulator *Cdk4* (Horsley et al, 2008; Oro, 2008). Conversely, sirtuin protein family member SIRT7, which is upregulated during the telogen-to-anagen transition, has been shown to briefly destabilize NFATC1, thus permitting the onset of anagen (Li et al, 2020).

Recent scRNAseq analyses have revealed that both *Foxc1* and *Nfatc1* reside throughout the murine anagen bulge (lower to upper) but exhibit highest expression in the upper region (Chovatiya et al, 2021) (weak expression has also been reported in the human anagen bulge (Wu et al, 2022)). *Nfatc1* has also been found in the telogen upper bulge (Joost et al, 2016) (region III, data not shown), confirming earlier IF analyses (Horsley et al, 2008).

### GLI1

GLI1 is a transcription factor involved in Hedgehog signaling, a major developmental pathway in which the SHH ligand binds to the receptor PATCHED1, promoting GLI1 induction of downstream gene targets, including *Gli1*. The HFSC populations expressing GLI1 during telogen include a lower bulge population along with HG residents that respond to SHH-expressing cells in the underlying DP (Figure 1a) and a second population within the upper bulge adjacent to a cutaneous nerve SHH source (Abe and Tanaka, 2017; Brownell et al, 2011). During follicular downgrowth, GLI1 is lost from the lower bulge as the DP, the SHH source, moves further away (Avigad Laron et al, 2018), whereas GLI1 expression is maintained in the upper bulge throughout cycling (Brownell et al, 2011). It was postulated that SHH signaling in the lower bulge is requisite for transient HFSC regeneration and maintenance (Avigad Laron et al, 2018), although long-term labeling studies suggested that these cells are involved in follicle downgrowth during anagen and are subsequently lost (Brownell et al, 2011). A role for SHH signaling in the upper bulge remains unclear for hair maintenance, although this function is requisite for participation of these cells in sustained wound healing (Brownell et al, 2011).

*Gli1* transcription has been verified in HFSCs residing in telogen LGR6+ region III of the upper bulge (Joost et al, 2016) (Table 1 and Figure 1b: compartment “III”). GLI1 and LGR6 have independently defined an upper bulge SC population capable of upper follicle maintenance and endowed with long-term wound-healing properties (Brownell et al, 2011; Snippert et al, 2010), suggesting that a single upper bulge population undertakes these functions.

### DAB2

DAB2 (disabled-2) is a clathrin and cargo binding endocytic adaptor protein shown to have diverse roles in cell signaling. Roy et al (2024) found upregulated expression of this protein in the telogen bulge and HG. Related studies using conditional K14CreER^+/−^*Dab2*^*fl/fl*^ (*Dab2-*conditional KO) mice revealed delayed hair cycling and reduced HFSC proliferation. Mechanistic studies showed that DAB2 bound WNT activity modulator DVL2 (dishevilled-2) to promote WNT-mediated proliferation (Roy et al, 2024). Although scRNAseq results remain limited, this transcript has been identified alongside other telogen upper bulge transcripts, including *Lgr5* and *Gli1* in pseudospatial analysis (Joost et al, 2016).

## Markers that Define Human HFSCs

Only a few studies have thoroughly investigated human HFSC markers. In this section, we provide information about a subset of recently validated/identified markers. Figure 1b and Table 1 provide additional information.

### CD200

This membrane glycoprotein, also known as OX-2, is a member of the Ig superfamily and has been reported to exert inhibitory effects on immune cells bearing the CD200R1 receptor (Vaine and Soberman, 2014). It was identified as a putative HFSC marker using laser-capture microdissection and microarray transcriptome profiling (Ohyama et al, 2006). Sorted CD200+ HF cells demonstrated high colony-forming efficiency in clonogenic assays, suggesting that CD200 might identify a true HFSC population (Ohyama et al, 2006). These results were subsequently corroborated by those from Inoue et al (2009). Because human HF transplants are well-tolerated (Bertolini et al, 2020), CD200 has been proposed as an effector of HF immune tolerance (Rosenblum et al, 2004). Recent studies have confirmed *CD200* expression in human bulge by scRNAseq (Ober-Reynolds et al, 2023) and IF analysis (Wu et al, 2022). *CD200R1* expression remains unknown.

### Vimentin

Vimentin (VIM) is a type III intermediate filament cytoskeletal protein that is typically expressed in mesenchymal cells, including HF DP (Kuburich et al, 2022). It is often associated with epithelial-to-mesenchymal transition events. Wu et al (2022) made the surprising discovery that *VIM* gene expression is found in human HFSCs but absent from murine skin. IF of human HFs confirmed protein expression in some upper HF bulge K15+ cells but not in the eyelid IFE or scalp dermis, indicating that this is primarily a feature of HFSCs (Wu et al, 2022) (Table 1 and Figure 1b: compartment “S1”).

### Dystonin

Dystonin (DST) is a member of the plakin family of adhesion junction plaque proteins. Several isoforms of DST are expressed in epithelium and involved in anchoring keratin-containing intermediate filaments to hemidesmosomes (Künzli et al, 2016). Its expression has been detected in the human bulge region, where it colocalizes with COL17A1 and K15 expression (Wu et al, 2022) (Table 1 and Figure 1b). Of note, the colocalization of DST with K15, which is a structural component of hemidesmosomes expressed by bulge HFSCs as well as its colocalization with COL17A1, whose expression changes with age, positions this marker as a suitable candidate for future studies.

### CXCL14

CXCL14 (C-X-C motif chemokine ligand 14) is a chemokine with documented functions as an immune cell chemoattractant during inflammation. A recent study has documented functions of this chemokine in neural development (Zhang et al, 2024). In situ analyses revealed *Cxcl14* expression in the murine HF bulge (Ojeda et al, 2013), and scRNAseq analyses from 2 groups confirm its expression in human HFSCs (Takahashi et al, 2020; Wu et al, 2022) (Table 1). Its role in the bulge remains unknown.

### Follistatin

Follistatin is a highly ubiquitous glycoprotein involved in activin and other TGFB superfamily member regulation (Hinck et al, 2016). Its expression in human HFSCs has been confirmed by RNA-sequencing and scRNAseq studies (Ohyama et al, 2006; Wu et al, 2022) (Table 1 and Figure 1b: compartment “S3”).

## miRNAs as HFSC Markers

miRNAs are small (approximately 21–23 bases) single-stranded RNAs that bind to the transcripts of target genes, preventing their translation. Previous studies knocking out miRNA processors *Droshe* or *Dicer* in epithelium provided compelling evidence for their importance to hair cycling (Andl et al, 2006; Teta et al, 2012). Recent scRNAseq analyses focusing on specific HFSC miRNAs during the hair cycle has brought to light important information about their temporal expression. Genetic and pharmacologic experiments have addressed their function(s) to regulate downstream targets and their activity. Given that the expression of miRNAs is managed during the hair cycle, they may be considered phase-specific HFSC marker candidates. Loss or increased expression of some of these miRNAs has been reported during aging (section on changes in the expression of markers with aging provides more details).

### Mouse

Table 2 provide the details.

**Table 2:** miRNAs as HFSC Markers

miRNAs	miRNA-29a/b (Ge et al, 2019)	miRNA-205 (Wang et al, 2023)	miRNA-148a (Pickup et al, 2023)	miRNA-31 (Yu et al, 2021)	miRNA-31 (Yu et al, 2021)	miRNA-324-3p (Mohammadi et al, 2021)
Species	Mouse	Mouse	Mouse	Mouse	Human	Human
Expression	Telogen	Telogen–anagen transition	Telogen	Telogen		Anagen compares alopecia with normal
Targets	*Lrp6*, *Ctnnb1* (WNT signaling components)	Actin cytoskeleton, adhesion, junctions, *Piezo1*	*Rock1*, E*lf5*	*Clock*		
Function	Inhibit WNT, BMP, division	Reduce stiffness	Presumed actomyosin cytoskeleton regulates contractile force	Activates MAPK pathway for HFSC depletion		Regulation of MAPK, TGFβ signaling
Location	HFSC	HFSC, HG	HFSC	Highest in HFSC		HFSC
Aging		Expression reduced		Expression increased	Expression increased	Lost in alopecia

Abbreviations: HFSC, hair follicle stem cell; HG, hair germ; miRNA, microRNA.

#### miR-29a/b1

This miRNA shows highest expression in HFSCs during telogen (Ge et al, 2019). Conditional overexpression of miR-29a/b1 using doxycycline (DOX)-inducible *K14-rtTA*/*TRE-*miR-29a/b1 double transgenic mice revealed downregulation of LEF1, AXIN2 (proteins indicative of active WNT signaling), and BMP signaling. Hair cycling progression was halted, and eventual hair loss was observed (Ge et al, 2019).

These findings suggested that miR-29a/b1 inhibits WNT and BMP signaling during normal telogen to maintain HFSCs in a dormant state. The loss of miR-29a/b1 thus marks the transition to anagen. Known targets include *Lrp6*, *Ctnnb1*, and *Bmpr1a.*

#### miR-24

This miRNA has been credited with a role in HG progenitor quiescence during telogen. We have included this miRNA in this review because it likely also impacts lower bulge populations.

An analysis of cycling HFs revealed high miR-24 expression in telogen and anagen bulge, whereas HG exhibited reduced miR-24 expression during telogen, which was gradually lost in down-growing matrix during anagen progression (Liu et al, 2021). This expression pattern suggested a role for miR-24 in telogen HG quiescence (Liu et al, 2021). Conditional miR-24 overexpression models revealed delayed HF anagen onset, whereas conditional KOs exhibited increased hair growth, suggestive of premature HG activation. The authors found that *Plk3*, which is considered to have a putative role in cell cycle progression, was a direct target of miR-24. They hypothesized that negative regulation of *Plk3* during telogen promotes HG quiescence.

#### miRNA-205

This miRNA arose in a study investigating bulge and HG cell stiffness during the hair cycle to understand how cell mechanics can govern hair cycling (Wang et al, 2023). It was found that HFSCs are stiffer than HG progenitors, especially during telogen, which correlates with increased F-actin levels. Further work showed that miRNA-205, whose expression induced widespread downregulation of targeted genes involved in actin cytoskeleton, was highly expressed in bulge and HG during the telogen-to-anagen transition.

The authors postulated that expression of miRNA-205 during the critical telogen-to-anagen phase reduces cell stiffness to permit cell division. However, this effect was primarily observed in the HG, where the stiffness threshold is lowest.

#### miRNA-148a

Its expression has been observed in CD34+K15+ HFSCs primarily during telogen (Pickup et al, 2023). Administration of an antisense inhibitor during telogen promoted anagen induction, suggesting the importance of this miRNA in ensuring telogen quiescence. The authors revealed that the target genes of miRNA-148a include *Rock1*, a major effector of small GTPase RHOA and a regulator of the actomyosin cytoskeleton to promote contractile force generation, and *Elf5*, an epithelium-specific subclass of the E26 transformation-specific transcription factor family.

#### miRNA-31

This miRNA has been shown to be upregulated in aged telogen HFSCs. Its induction leads to a variety of age-related HFSC changes, which are discussed below in the Teat section on aging (Yu et al, 2021).

### Human

Details are provided in Table 2.

#### miR-324-3p

This miRNA was discovered in a study by Mohammadi et al (2021), who compared human HFSCs (K15+CD200+) from normal with those from androgenetic alopecia HFs (Mohammadi et al, 2021). Results are discussed below in the section on aging.

## Changes in the Expression of Markers with Aging

Age-related alopecia is a prevalent condition affecting both sexes worldwide (Harrison and Bergfeld, 2009; Matsumura et al, 2021; Paus and Cotsarelis, 1999). Although the causes remain elusive, it has been clearly demonstrated that hair cycling slows during aging, and HFs remain in telogen for longer periods as they age (Chen et al, 2014; Keyes et al, 2013). Furthermore, regions of active hair growth progressively shrink, which might indicate an environmentally driven contribution (Chen et al, 2014). Ultimately, HFs undergo miniaturization, exemplified by reduced hair shaft diameter, DP size reduction, and loss of sebaceous glands and the HF infundibular region (Baltenneck et al, 2022; Rittié and Fisher, 2015; Williams et al, 2020; Xie et al, 2022). Ultimately, miniaturized follicles give rise to vellus hair and are eventually lost (Reygagne and de Lacharrière, 2005).

Because alopecia is irrevocably tied to hair cycle changes (Qi and Garza, 2014), age-related changes in HFSCs have been scrutinized. Surprisingly, numerous studies examining both murine and human HFSCs prior to and after miniaturization commonly agree that HFSC numbers and lineage identity remain the same as in youth until miniaturization begins (Garza et al, 2011; Ge et al, 2020; Giangreco et al, 2008; Keyes et al, 2013; Matsumura et al, 2021; Rittié et al, 2009). Therefore, the induction of this phenomenon cannot be directly tied to loss of HFSCs but may rather involve alteration of their functionalities.

**Figure 2:**
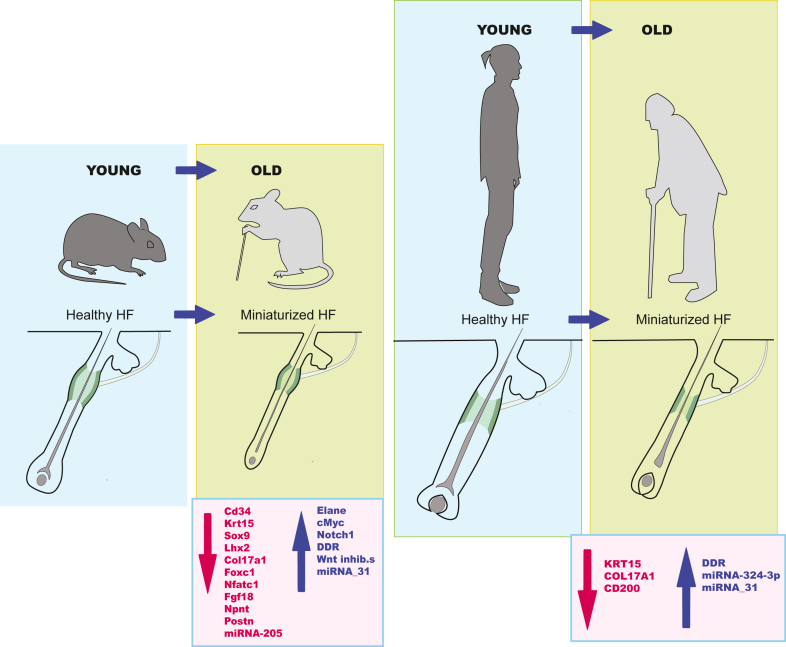
**Markers that change transcriptional expression during aging.** Left panels: mouse. Right panels: human. DDR, DNA damage response; HF, hair follicle.

Studies focusing on changes in HFSC gene expression just prior to miniaturization have confirmed basic aging features such as prolonged quiescence and proposed mechanisms for age-related alopecia. In this section, we will examine some of these mechanisms, focusing on how changes in known marker expression can drive alopecia. Markers that arise with aging are also addressed (Figure 2 provides a summary of these findings).

### Mouse

#### WNT inhibitors to promote long-term HFSC quiescence

WNT activation is a crucial first step in HFSC activation during anagen onset. To ask whether this basic signaling pathway was perturbed with aging, Chen et al (2014) examined canonical WNT ligands and the WNT effector CTNNB1 (catenin beta-1) and found similar levels in both young and aged HFs. However, aged HFs also expressed significantly increased levels of WNT inhibitors DKK1 and SFRP4, indicating a shift in the balance between SC activating and inhibitory signals toward inhibition in older mice.

#### NFATC1/SIRT7 to promote long-term HFSC quiescence

In earlier studies, Keyes et al (2013) found that aged HFSCs are ‘sluggish’ in progressing to anagen onset, which they attributed to increased levels of HFSC quiescence factor NFATC1 (details are provided earlier). However, more recent results carefully examining gene expression by scRNAseq suggested that *Nfatc1* may remain stable or reduce in transcriptional levels in aged HFSCs (Ge et al, 2020; Zhang et al, 2021) (details are provided below). It is intriguing that deacetylase SIRT7, with a role in deacetylating NFATC1 protein for its degradation, is downregulated in the aging HF bulge (Li et al, 2020). Thus, stable rather than elevated levels of NFATC1 protein during anagen onset in aged HFSCs may help to explain this phenotype at least in part. In support of this, overexpression of SIRT7 in aged HFSCs was shown to promote hair growth (Li et al, 2020).

#### NFATC1/FOXC1 loss for HFSC escape

Recent results from Zhang et al (2021) suggest that loss of *Nfatc1* and *Foxc1* during aging may actually promote HFSC movement out of the bulge, ultimately resulting in bulge size reduction and HF miniaturization. In this interesting study, 6% K14-H2bGFP bulge HFSCs from old (aged 24 months) mice tracked by live imaging were shown to migrate out of the bulge. This phenomenon was not observed in young animals. scRNAseq revealed decreased *Foxc1* and target *Nfatc1* transcription in 24-month telogen HFSCs compared with p53 telogen HFSCs, which was accompanied by lower expression of cell adhesion genes (Zhang et al, 2021). *Foxc1* × *Nfatc1* double KOs were required to generate a robust phenotype, suggesting additional unique nonoverlapping roles for these genes (details are provided in the section on NPNT).

#### COL17A1 loss and ELANE upregulation for HFSC escape

Matsumura et al (2016) compared fluorescence-tagged HFSCs from young with those from aged *K15*-CrePR;CAGCAT-EGFP mice and noted that HFSCs from aged mice had the propensity for migrating out of the bulge and up into the IFE during anagen (Matsumura et al, 2016). Migration coincided with changes in marker expression shifting toward an IFE epithelial phenotype. This phenomenon was not observed in young mice, where HFSCs either remained in the bulge or participated in anagen downgrowth as expected. scRNAseq examination of aged HFSC genes revealed a reduction in the expression of HFSC markers *Sox9*, *Col17a1*, and *Lhx2* prior to miniaturization as well as upregulation of a gene signature corresponding to DNA damage response (DDR) (Matsumura et al, 2016).

To ask whether their results might be explained by a reduction in HFSC adhesion with loss of COL17A1, they focused on serine protease ELANE. Undetectable in young HFs, ELANE was found to be upregulated in aged HFSCs, and experimentation revealed that induction of DDR by infrared radiation induced high expression of ELANE with concomitant loss of COL17A1 in young HFs. They concluded that ELANE in aged HFSCs induced proteolysis of COL17A1, resulting in destabilization of HFSCs within the bulge and their subsequent release toward the IFE. Targeted overexpression of *Col17a1* in transgenic mice resulted in the partial rescue of aged HFs as evidenced by fewer miniaturized HFs (Matsumura et al, 2016).

Aged human miniaturized HFSCs showed significant decreases in *K15*, *CD200*, and *Col17a1* and increased H2AX foci indicative of DDR, and occasional IFE marker expression was also observed in aged bulge HFSCs, all suggesting a similar phenomenon in humans.

#### Dysregulation of ECM and ECM-related genes

Ge et al (2020) undertook scRNAseq analyses to perform a careful survey of established HFSC markers in young (aged 2 months) and aged (2 years) telogen HFSCs and concluded that most examined markers exhibited persistent albeit reduced expression over time. Nevertheless, in mice, the aged bulge did exhibit a clear reduction in several ECM and ECM-associated genes, including *Col1a1*, *Col4* family members, *Emilin3*, *Pcolce2*, and *Spon2*, which the authors postulated might be important for preserving bulge structural integrity. *Fgf18*, a key FOXP1 target and important regulator of HFSC quiescence, was also downmodulated (Leishman et al, 2013) (more details are provided in the section on NPNT below).

In addition, the aged telogen bulge exhibited several significant physical changes, including frequent absence of the club hair and therefore contribution from the club hair bulge, sensory nerve dislocation, and apparent detachment of the APM from the bulge, correlating with reduced expression of NPNT (details are provided below). Overall, the authors concluded that aged telogen HFSCs show remarkably high fidelity for the original SC state. They demonstrated this in hair reconstitution assays in which young dermis mixed with old HFSCs resulted in the generation of HFs, suggesting that they are essentially capable of rejuvenation (Ge et al, 2020).

#### APM–synaptic nerve–bulge axis

NPNT is a protein required for APM attachment to the bulge whose gene expression has been confirmed in scRNAseq analyses during anagen and telogen (details provided earlier). Several studies have reported its downregulation with aging, concurrent with loss of APM attachment to the bulge (Ge et al, 2020; Zhang et al, 2021). Interestingly, an APM–sympathetic nerve–bulge axis has recently been described, which is crucial for HFSC entry into anagen (Shwartz et al, 2020). In this axis, sympathetic nerves supported by the APM form synapse connections with HFSCs and secrete norepinephrine, which is bound by adrenergic receptor ADRB2 on HFSCs. Loss of either associating nerves or of ADRB2 on HFSCs resulted in delayed anagen entry. Furthermore, RNA-sequencing analysis of *Adrb2*-KO HFSCs revealed upregulated transcription of quiescence genes *Foxp1* and *Fgf18*, indicating that this axis somehow negatively modulates quiescence genes to promote cycle entry (Shwartz et al, 2020).

Thus, loss of the APM connection in aged bulge may point to a mechanism for the observed profound quiescence of these cells.

### Human

To date, few scRNAseq comparisons exist to ask detailed questions about changes in human HFSCs with aging. Consequently, most analyses are carried out in mice, and some results are correlated in humans (Figure 2).

Wu et al (2022) undertook a limited scRNAseq comparison across different age groups of women. HFs dissected from individuals aged 18, 31, 59, and 62 years were compared for matching changes across pairs (F18–F59 and F32–F62), but ultimately, the authors concluded that no significant overlap could be identified, thereby underscoring the challenge posed by individual-to-individual variation.

They also compared scRNAseq results across pigmented versus unpigmented (gray) hairs within several individuals (Fe31bl vs Fe31w, Fe62bl vs Fe62w) to identify features common to both groups. Because hair graying, due to loss or dysfunction of hair melanocytes (Commo et al, 2004), is an important aging phenomenon, it can provide relevant information about HFSCs, which play essential roles in MelSC quiescence and activation within the bulge (Paus et al, 2024). Elevated TP53 pathway genes were observed in the white HFSC cluster in both groups, reminiscent of DDR upregulation observed in murine aged HFSC aging (Matsumura et al, 2016), suggesting some commonality between HFSC aging and hair graying. Larger cohort studies will provide important information about this interesting observation.

### miRNAs (mouse and human)

Several miRNAs have also been investigated for changes with aging (Table 2 provide the summary). miRNA-205 function has been mapped to anagen onset where it reduces HFSC contractility for cell cycle entry (Wang et al, 2023). Results showed that it was downregulated in old murine HFs (aged 19 months) as was its transcriptional regulator DeltaN-TP63. Induction of *K14*-driven miRNA-205 in old HFSCs promoted rapid anagen onset, suggesting that this miRNA alone has a potent capability that appears independent of external factors (Wang et al, 2023).

In contrast, miRNA-31 may be considered a valid aging marker because it is highly upregulated in aged (22 months) telogen HFSCs (Yu et al, 2021). *K14*-driven miRNA-31 overexpression models demonstrated an aging phenotype after 5 months of DOX treatment, including baldness; HF miniaturization; and loss of HFSC markers K15, SOX9, CD34, and COL17A1. Conversely, KO of miRNA-31 suppressed early HF aging in an irradiation aging model (Yu et al, 2021). An important miRNA-31 target proved to be circadian rhythm gene *Clock*, a gene known to play important roles in HF anagen progression (Niu et al, 2023). Because the circadian clock is known to have reduced impact with aging, miRNA-31 is likely involved. mRNA-31 is upregulated in aged human skin as well as in HFs, suggesting a broader role in humans (Yu et al, 2021).

In this interesting study comparing human miRNAs from normal with those from androgenic alopecia (AGA) HFSCs, Mohammadi et al (2021) found a potential human HFSC marker in mRNA-324-3p. AGA, which affects both men and women, is commonly associated with increased sensitivity to androgens, although more recent data suggest a strong link to other aging-related changes (Deng et al, 2023; Ntshingila et al, 2023). In comparing transcriptomes from K15+CD200+ HFSCs from normal hair (including normal patient hair) with HFSCs from miniaturized HFs, they found 82 miRNAs specifically upregulated in normal HFSCs. High differential expression of miRNA-324-3p in particular was noted. Preliminary results using culture systems suggested a TGFB-related role for miRNA-324-3p in keratinocyte differentiation and migration (Mohammadi et al, 2021). A more detailed examination of this miRNA and others discovered by Mohammadi et al (2021) will likely provide some candidate markers and insights into AGA development.

## Conclusion

In summary, candidate markers of HFSCs have been identified within groups of molecules corresponding to a broad panel of biological characteristics and functions. Concerning the protein-coding genome, a panel of markers consisting of surface proteins suitable for viable cell sorting is available to further refine the separation of the so-called HFSC population into functionally distinct subpopulations. Nonexhaustively, intracellular proteins referenced as HFSC markers comprise transcription factors, cytoskeleton components, and various signaling molecules, available for purposes such as HFSC follow-up, tracking, or exploration of SC biology. The search for HFSC markers also extends into the field of noncoding RNAs, with data available on miRNAs. Other classes, notably long noncoding RNAs, certainly offer promising research fields.

Further knowledge of defined and yet-to-be-discovered HFSC molecular markers and their changes with aging will be essential for making meaningful progress toward enhancing hair longevity. This will require additional genome-wide profiling approaches, including bulk RNA sequencing on well-defined samples, and scRNAseq approaches, especially in humans. Spatial transcriptomics will provide detailed information about cell–cell locations (and their changes with aging). In addition, computational approaches integrate information about coding and noncoding regions (combined scRNAseq and Assay for Transposase-Accessible Chromatin using sequencing for example), as performed by Ober-Reynolds et al (2023). All these data contribute to the development of the very useful “Human Cell Atlas” data portal initiative, the “Skin Network” (coordinated by M. Kasper, M. Plikus, and F. Watt [Almet et al, 2023]) (https://data.humancellatlas.org/hca-bio-networks/skin/datasets or https://skingenes.biochem.uci.edu/), which will certainly contribute to advances in this field.

## ORCIDs

Theebah Sellathurai: http://orcid.org/0009-0007-7492-9583

Denise L. Gay: http://orcid.org/0000-0001-7340-8427

Stéphane Commo: http://orcid.org/0000-0002-8363-0299

Gilles Lemaître: http://orcid.org/0000-0002-0898-1582

Nicolas O. Fortunel: http://orcid.org/0000-0001-8702-247X

## Conflict of Interest

TS and SC are L’OREAL employees engaged in research activities. The remaining authors state no conflict of interest.
